# Machine Learning‐Based Prediction of Life‐Threatening Complications During Hemodialysis in Hospitalized Patients With Poor General Conditions

**DOI:** 10.1111/aor.70008

**Published:** 2025-09-20

**Authors:** Naotaka Kato, Takeshi Goto, Tomoyuki Ohira, Hirotaka Kinoshita, Kugo Kurokawa, Kouhei Naganuma, Chikako Ohminato, Junko Ogasawara, Shingo Hatakeyama, Yoshihiro Sasaki, Kazuyoshi Hirota, Chikara Ohyama

**Affiliations:** ^1^ Department of Clinical Engineering Hirosaki University School of Medicine and Hospital Hirosaki Japan; ^2^ Department of Anesthesiology Hirosaki University Graduate School of Medicine Hirosaki Japan; ^3^ Department of Urology Hirosaki University Graduate School of Medicine Hirosaki Japan; ^4^ Department of Medical Informatics Hirosaki University Graduate School of Medicine Hirosaki Japan; ^5^ Departments of Perioperative Stress Management Hirosaki University Graduate School of Medicine Hirosaki Japan; ^6^ Department of Advanced Transplant and Regenerative Medicine Hirosaki University Graduate School of Medicine Hirosaki Japan

## Abstract

**Background:**

Patients undergoing hemodialysis (HD) face a significantly elevated risk of cardiovascular mortality, with sudden events during treatment posing a critical threat to survival. These risks are particularly pronounced in high‐risk populations, such as patients recovering from cardiovascular surgery or those being treated for sepsis. Therefore, the development of effective preventive strategies is essential for improving patient outcomes. This study aimed to develop a machine learning model that uses pretreatment patient characteristics to predict sudden adverse events during HD and within 24 h after treatment in high‐risk inpatients at acute care hospitals.

**Methods:**

His retrospective study analyzed data from 739 patients who underwent HD at Hirosaki University Hospital between 2018 and 2021. Sudden events were defined as fatal arrhythmia, refractory intradialytic hypotension, or respiratory arrest. A logistic regression model was constructed using backward stepwise selection from 51 patient characteristics (demographic data, clinical parameters, laboratory data, and HD‐related information).

**Results:**

Among the 739 patients, 17 (2.3%) experienced sudden events. The model identified 23 pre‐HD covariates and achieved an area under the receiver operating characteristic curve (AUC) of 0.889. Key covariates included emergency hospitalization (present in 71% of patients with sudden events), recent surgery (76%), shorter HD history, elevated pre‐HD heart rate, lower serum albumin levels, and higher C‐reactive protein concentrations.

**Conclusions:**

Our model enables the early identification of high‐risk inpatients receiving hemodialysis using pre‐dialysis data, thereby supporting timely clinical interventions, optimized resource allocation, and improved patient safety.

## Graphical abstract

A machine learning model using predialysis data predicted sudden events during or after hemodialysis with high accuracy (auROC: 0.889). The key predictors included emergency hospitalization, recent surgery, high heart rate, low albumin levels, and high CRP. This model enables the early identification of high‐risk patients and supports timely interventions.

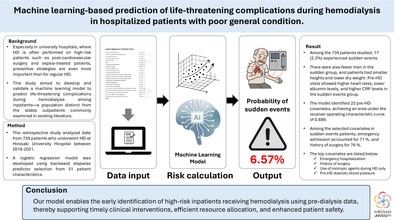

## 1 Background

Nearly four million individuals worldwide undergo kidney replacement therapy, with hemodialysis (HD) being the most commonly used modality [1]. Approximately two‐thirds of HD patients have cardiovascular disease (CVD), leading to a significant symptom burden and reduced quality of life. In Japan alone, more than 340,000 patients receive HD. According to a 2023 report by the Japanese Society for Dialysis Therapy, 27.2% of HD patients die from cardiovascular causes, and 0.8% die suddenly—highlighting the substantial mortality burden in this population [2].

Sudden cardiac death (SCD) during HD is a critical and highly undesirable event. Although relatively rare, approximately 60% of patients who experience cardiac arrest during HD die within 48 h [3]. Proposed mechanisms include fluctuations in circulating blood volume and electrolyte imbalances during HD [4]. Another known risk factor is intradialytic hypotension (IDH), defined as a symptomatic decrease in systolic blood pressure of ≥ 20 mmHg or a drop in mean arterial pressure of ≥ 10 mmHg during HD [5]. IDH is more common among patients with diabetes, CVD, left ventricular hypertrophy, advanced age, and excessive interdialytic weight gain [6]. Its prevalence has been reported as 31% among inpatients and 36% in intensive care unit (ICU) patients [7]. Panocchia et al. found that in‐hospital mortality reaches 31.3% among patients who initiate HD emergently without prior preparation [8]. While patients with multiple comorbidities and poor general health are considered at higher risk for SCD, predictive factors remain poorly defined, making accurate risk stratification challenging.

To address these challenges, machine learning has been increasingly applied to predict life‐threatening complications in patients receiving HD. Several studies have developed machine learning models to predict SCD, IDH, and early mortality in outpatient HD populations, demonstrating promising predictive performance [9, 10, 11, 12, 13]. However, these models have primarily focused on stable outpatients. To date, no machine learning‐based predictive models have been developed specifically for inpatients receiving HD in acute care settings, where patients often present with complex clinical histories, such as recent cardiovascular surgery or sepsis following ICU admission.

This study aimed to develop a machine learning model using pre‐treatment patient characteristics to predict sudden adverse events during HD and within 24 h after treatment in high‐risk inpatients at acute care hospitals. Compared to outpatients, inpatients undergoing HD often exhibit more complex and unstable conditions. Therefore, predictive models tailored to this population may support improved prevention strategies and enhance patient safety.

## 2 Methods

### 2.1 Patients and Study Approval

We enrolled 739 patients who underwent hemodialysis (HD) at Hirosaki University Hospital (Hirosaki, Japan) between January 1, 2018, and December 31, 2021. Our institution is not a chronic dialysis facility but rather a center equipped with an acute dialysis unit designed to support surgical procedures and other acute medical needs. Standard HD procedures using hollow fiber membrane dialyzers were employed.

All patients were included without the application of exclusion criteria. This retrospective, observational, single‐center study was conducted in accordance with the Strengthening the Reporting of Observational Studies in Epidemiology (STROBE) guidelines and was approved by the Ethics Committee of the Hirosaki University Graduate School of Medicine (Approval No. 2024–029). The requirement for written informed consent was waived by the Ethics Committee.

### 2.2 Data Collection and Definition of Sudden Events

The dataset consisted of 51 characteristics of 739 patients who underwent HD in the acute dialysis unit between 2018 and 2021. This included demographic data (age, dry weight, and ejection fraction), clinical parameters (initial pre‐blood pressure and initial pre‐heart rate), and laboratory data (e, g. hemoglobin, CRP, lymphocytes), and HD‐related information (e.g., treatment setting: HD, hemodiafiltration, extracorporeal ultrafiltration, vascular access). The sudden event group included patients with fatal arrhythmia, IDH refractory to both fluid resuscitation and vasopressor therapy during HD, and respiratory arrest.

### 2.3 Backward Stepwise Predictor Selection

A backward stepwise approach was employed for feature selection in the development of a binary logistic regression model using the Python software (version 3.8; www.python.org; Python Software Foundation, Wilmington, DE, USA) with the scikit‐learn library. The process begins with a comprehensive model that incorporates all covariates. It then iteratively eliminates the least significant covariates one at a time until the model includes only a single covariate. The selection of covariates for removal at each step was determined by maximizing the mean validation area under the receiver operating characteristic curve (validation auROC) obtained through 10 iterations of threefold cross‐validation.

### 2.4 Calculation Process of Probability *p* (Occurrence∣*p*) in Logistic Regression

#### 2.4.1 Parameters of the Trained Model

A logistic regression model employs the sigmoid function, mathematically expressed as:

$$\begin{array}{c} \text{𝑝 (occurrence∣𝑋)}=\text{𝜎 (𝑧)}=1/\left(1+\exp\;\left(-z\right)\right) \end{array}$$

Here, the linear predictor (logit function) 𝑧 is defined as:

$$\begin{array}{c} \text{𝑧}=\text{𝑤0+𝑤1𝑥1+𝑤2𝑥2+⋯+𝑤𝑛𝑥𝑛} \end{array}$$

𝑥 = (𝑥1, 𝑥2, …, 𝑥𝑛) denotes the new input feature vector. 𝑤 = (𝑤1, 𝑤2, …, 𝑤𝑛) presents the trained weight coefficients. *w*0 is the bias (intercept).

#### 2.4.2 Scaling the New Input Data

Because the logistic regression model is trained on standardized features, it is imperative to apply the same StandardScaler transformation to the new input data Xnew to ensure consistency.

Xnew,scaled=(Xnew‐𝜇)/𝜎

where 𝜇 and 𝜎 represent the mean and standard deviation of the training data.

#### 2.4.3 Computing the Linear Predictor

For the standardized input data 𝑋new, scaled, the linear predictor Znew is computed using the trained weight vector and bias term:

$$\begin{array}{c} \text{𝑝}\;\left(\text{occurrence}|\text{Znew}\right)=1/\left(1+\exp\;\left(-\text{Znew}\right)\right) \end{array}$$

**FIGURE 1:**
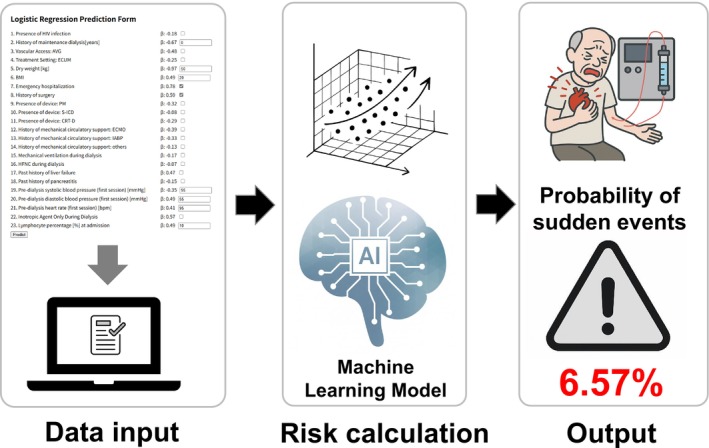
Illustration of a clinical application for estimating sudden events probability. [Color figure can be viewed at wileyonlinelibrary.com]

This probability value quantifies the likelihood of intradialytic hypotension during HD, with higher values indicating an elevated risk. The equation presented above forms the basis for a clinical application that enables the real‐time estimation of the probability of life‐threatening complications during HD. To simplify the complex calculations in clinical practice, an estimation process was conducted following the procedure illustrated in Figure 1, which facilitates the assessment of the risk of sudden events.

### 2.5 Statistical Analysis

Patient data are presented as median (25th–75th percentiles) or number (percentage of each group). To compare patient characteristics between sudden group and non‐sudden group, we used Fisher's exact test to examine the significance of between‐group differences for categorical variables and the Wilcoxon rank‐sum test for continuous variables. All statistical analyses were performed using SPSS Statistics for Windows (version 21.0; IBM Corp., Armonk, NY, USA) and R software version 4.2.2 (R Foundation for Statistical Computing, Vienna, Austria). Statistical significance was set at *p* < 0.05 for all tests.

## 3 Results

The remaining 739 patients were included in the final analysis. Of the 739 enrolled patients, 17 (2.3%) were assigned to the sudden group.

### 3.1 Patient Characteristics

Intergroup comparisons were performed between the sudden and nonsudden event groups (Table 1). More patients in the sudden group were on HD for less than 1 year than those in the non‐sudden group and were on HD for shorter periods. Compared to the non‐sudden group, the sudden group had a higher rate of emergency hospitalization, end‐of‐life care management, and liver failure in the sudden group. There were also fewer men in the sudden group, and the patients were shorter and had a lower dry weight. Pre‐HD vitals showed higher heart rates, lower albumin levels, and higher CRP levels in the sudden group.

**TABLE 1:** Dataset comprises 51 characteristics collected from 739 patients.

	Overall	Sudden events group	Not sudden events group	*p*	Odds ratio
Total patient number [%]	738	0.2 (17)	99.8 (721)		
Previously initiated HD patient (< 1 years) [%]	69 (510)	41 (7)	30 (503)	0.016	
Patient characteristics					
Gender (male)	68 (505)	35 (6)	68 [499]	< 0.01	0.243
Age, mean [SD]	67 [57–74]	70 [62.5–78.5]	67 [57–74]	0.22	
Height [cm], mean [SD]	162 [154–169]	154 [148–160]	162 [153–169]	< 0.01	
Dry weight [kg], mean [SD]	58 [50–68]	53 [42–59]	58 [50–68]	0.024	
BMI, mean [SD]	22.1 [19.8–25.3]	21.2 [18.6–25.0]	22.2 [19.8–25.3]	0.399	
Maintenance HD history [years], mean [SD]	2.4 [0–7.1]	0 [0–1.9]	2.5 [0–7.3]	< 0.01	
Ejection fraction [%]	0 [0–57.4]	0 [0–61.1]	0 [0–57.5]	0.845	
Initial pre‐HD systolic blood pressure (first session) [mmHg], mean [SD]	135 [117–155]	132 [112–143]	135 [117–156]	0.231	
Initial pre‐HD diastolic blood pressure (first session) [mmHg], mean [SD]	74 [64–85]	80 [57–86]	74 [64–85]	0.711	
Initial pre‐HD heart rate (first session) [bpm], mean [SD]	77 [67–86]	80 [57–86]	76 [67–86]	< 0.01	
Use of inotropic agents: during HD only [%]	4 (32)	1 (4)	4 (28)	< 0.01	7.567
Use of inotropic agents: also in the ward [%]	3 (19)	0 (2)	2 (17)	0.067	5.492
Hemoglobin value at admission [mg/dL], mean [SD]	11.1 [9.4–12.4]	9 [8.6–12.1]	11.1 [9.4–12.4]	0.095	
Albumin value at admission [mg/dL], mean [SD]	3.3 [2.8–3.8]	2.6 [2.3–3.4]	3.3 [2.8–3.8]	< 0.01	
C‐reactive protein value at admission [mg/dL], mean [SD]	0.51 [0.09–3.24]	4.66 [0.85–8.25]	0.46 [0.09–3.12]	< 0.01	
Brain natriuretic peptide value at admission [pg/dL], mean [SD]	0 [0–198]	0 [0–375]	0 [0–191]	0.743	
Neutrophil percentage [%] at admission, mean [SD]	69.2 [8.29–79.5]	75.3 [0–85.8]	69.1 [8.47–79.2]	0.439	
Lymphocyte percentage [%] at admission, mean [SD]	13.6 [6–20]	10.2 [2.5–23.4]	13.6 [6.0–20.2]	0.629	
HD‐related information					
Vascular access					
Catheter [%]	32 (236)	1 (8)	31 (228)	0.193	1.92
AVF [%]	60 (441)	1 (7)	59 (434)	0.137	0.463
AVG [%]	4 (30)	0 (0)	4 (30)	1	0
Superficialized artery [%]	4 (31)	0 (2)	4 (29)	0.157	3.173
Treatment setting					
HD [%]	48 (354)	1 (10)	47 (344)	1	0.7
I‐HDF [%]	28 (205)	0 (2)	27 (203)	0.175	0.34
On‐line HDF [%]	22 (160)	1 (4)	21 (156)	0.463	1.564
Off‐line HDF [%]	1 (9)	0 (1)	1 (8)	0.19	5.541
ECUM [%F]	1 (6)	0 (0)	1 (6)	1	0
Past medical history					
Emergency hospitalization [%]	39 (286)	2 (12)	37 (274)	< 0.01	3.907
Presence of end‐of‐life management [%]	4 (30)	1 (5)	3 (25)	< 0.01	11.49
History of ICU/ER admission [%]	45 (330)	1 (9)	3 (321)	0.623	1.401
History of surgery [%]	67 (492)	2 (13)	65 (479)	0.448	1.64
Past history: liver failure [%]	3 (19)	0 (3)	2 (16)	< 0.01	9.361
Past history: pancreatitis [%]	1 (5)	0 (0)	1 (5)	1	0
Past history: diabetes mellitus [%]	42 (314)	1 (8)	41 (306)	0.805	1.205
Past history: cardiopulmonary arrest [%]	3 (25)	0 (1)	2 (24)	0.447	1.813
History of mechanical circulatory support					
ECMO [%]	3 (21)	0 (0)	3 (21)	1	0
IABP [%]	2 (14)	0 (0)	1 (14)	1	0
Others [%]	1 (4)	0 (0)	1 (4)	1	0
Respiratory support during HD					
Mechanical ventilation [%]	1 (6)	0 (0)	1 (6)	1	0
NPPV [%]	0 (2)	0 (0)	0 (2)	1	0
HFNC [%]	0 (2)	0 (0)	0 (2)	1	0
Mask/cannula [%]	16 (119)	1 (6)	15 (115)	0.045	2.8689
Presence of CIEDs					
PM [%]	2 (18)	0 (0)	2 (18)	1	0
TV‐ICD [%]	1 (5)	0 (1)	1 (4)	0.11	11.08
S‐ICD [%]	1 (5)	0 (0)	1 (5)	1	0
CRT‐D [%]	1 (10)	0 (0)	1 (10)	1	0
CRT‐P [%]	0 (0)	0 (0)	0 (0)	1	0
Presence of infection					
HBV [%]	8 (60)	0 (2)	8 (58)	0.641	1.523
HCV [%]	8 (56)	0 (1)	7 (55)	0.018	0.757
HIV [%]	1 (7)	0 (0)	1 (7)	1	0
Syphilis [%]	4 (27)	0 (3)	3 (24)	0.021	6.189

Abbreviations: AVF, arteriovenous fistula; AVG, arteriovenous graft; CIEDs, cardiac implantable electronic devices; CRT‐D, cardiac resynchronization therapy with defibrillator; CRT‐P, cardiac resynchronization therapy with pacemaker; ECMO, extracorporeal membrane oxygenation; ECUM, extracorporeal ultrafiltration method; HD, hemodialysis; HDF, hemodiafiltration; HFNC, high‐flow nasal cannula; IABP, intra‐aortic balloon pump; ICD, implantable cardioverter‐defibrillator; I‐HDF, intermittent HDF; NPPV, noninvasive positive pressure ventilation; PM, pacemaker; S‐ICD, subcutaneous ICD; TV‐ICD, transvenous‐ICD.

### 3.2 Characteristics of Patients With Sudden Events During HD

Table 2 shows the characteristics of the patients who experienced sudden events. Of these patients, 82% had IDH, and 88% required discontinuation of HD. Regarding outcomes, 47% of patients receiving HD died during hospitalization, and 75% of these were patients who had not previously been on HD.

**TABLE 2:** Characteristics of patients with sudden events during HD.

Patient No.	History of maintenance HD [years]	Purpose of hospitalization	HD‐inducing disease	History of ICU admissions	Oxygen therapy	Comorbidities	Sudden events symptoms	Treatment after a sudden event	Outcome
1	0	Acute exacerbation of cardiac failure	Diabetic Kidney disease	−	O_2_ Cannula 2 L/min	Old myocardial infarction	IDH, NSVT	Interruption HD Admission to ICU	Discharge
2	0	Acute aortic dissection	septic AKI	+	T‐piece 3 L/min	Hypoxic ischemic encephalopathy	IDH	Use of inotropic agents	Discharge
3	0	Acute aortic dissection	Septic AKI	+	O_2_ mask 6 L/min	Panhypopituitarism Hypertriglyceridemia	Shivering, Cyanosis	Interruption HD Admission to ICU	Discharge
4	13	Intracerebral hemorrhage	Diabetic kidney disease	+	−	Diabetes Hepatitis C	IDH	Interruption HD Use of inotropic agents	Death
5	2	Urinary tract infection	Diabetic kidney disease	+	O_2_ Cannula 2 L/min	B‐cell lymphoma	IDH	Change to ECUM Use of inotropic agents	Death
6	0	Intrahepatic cholangiocarcinoma Liver failure	Septic AKI	−	−	Diabetes Hypertension	IDH, Sinus tachycardia	Interruption Escalation of inotropic agents	Death
7	0	Cholangitis	Septic AKI	+	−	Cholangiocarcinoma, Liver failure	IDH	Interruption HD	Death
8	0	Uremia	Polycystic kidney	−	−	Chronic Lymphocytic Thyroiditis Rheumatoid Arthritis Paroxysmal atrial fibrillation	IDH	Interruption HD Escalation of inotropic agents	Death
9	0	Alveolar hemorrhage	ANCA‐associated vasculitis with renal injury	+	O_2_ mask4L/min	Cytomegalovirus enteritis Paroxysmal atrial fibrillation	IDH	Replenishing liquid	Death
10	1.8	Diverticular bleeding	Chronic glomerulonephritis	−	−	Hypertension Hyperlipidemia Angina pectoris	Vf	CPR → Admission to ICU	Discharge
11	0	Fulminant hepatitis	AKI	+	O_2_ Cannula 3 L/min	−	Pulseless electrical activity	CPR → Admission to ICU	Death
12	2	Intrapelvic tumor	CKD	−	−	Chronic heart failure Chronic lymphocytic thyroiditis Hypertension atrial fibrillation Diabetes	IDH	Use of inotropic agents	Discharge
13	0	Aortic graft infection	Septic AKI	+	−	Pyogenic spondylitis	IDH	CPR → Admission to ICU	Death
14	0.3	Pancytopenia aplastic anemia	Chronic glomerulonephritis	−	−	Graves' disease	IDH	Interruption HD Replenishing liquid	Discharge
15	5.3	Impaired consciousness	Nephrosclerosis	+	−	chronic heart failure	IDH	Interruption HD	Discharge
16	1.3	Follicular lymphoma	Unknown	−	−	Pancytopenia Myelodysplastic syndromes	IDH	Interruption HD	Discharge
17	0	ANCA‐associated vasculitis	ANCA‐associated vasculitis with renal injury	−	−	Prostate Cancer	IDH Impaired consciousness	Interruption HD Admission to ICU	Discharge

Abbreviations: AKI, acute kidney injury; ANCA, antineutrophil cytoplasmic antibody; CKD, Chronic kidney disease; CPR, cardiopulmonary resuscitation; HD, hemodialysis; IDH, intradialytic hypotension; NSVT, nonsustained ventricular tachycardia.

### 3.3 The Binary Model's Performance at Each Stage of the Backward Stepwise Predictor Selection Process

**FIGURE 2:**
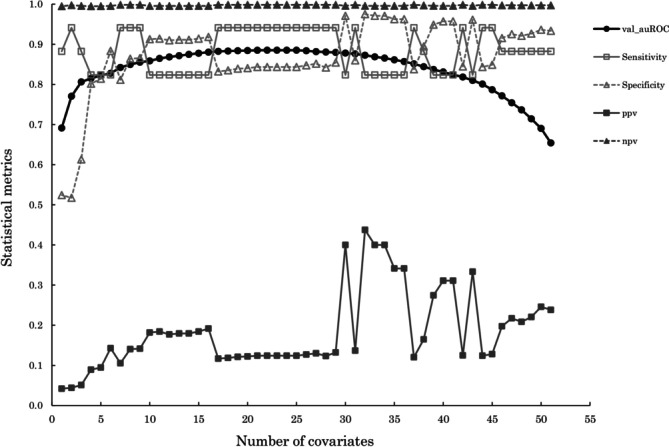
Binary model's performance at each stage of the backward stepwise predictor selection process.

Statistical indicators, including validation auROC, sensitivity, specificity, positive predictive value, and negative predictive value, were presented for the selected covariates and the covariate elimination process. The validation AUROC reached a peak of 0.889 when 23 covariates were selected (Figure 2).

The selected covariates and their coefficients are listed in descending order in Table 3. Among the selected covariates in sudden events patients, emergency admission accounted for 71%, and history of surgery for 76%.

**TABLE 3:** Covariates and coefficients for maximum receiver operating characteristic curve and parameters in patients with sudden events during HD.

Selected covariates with the maximal validation auROC	Patient No.																	Coefficients
	1	2	3	4	5	6	7	8	9	10	11	12	13	14	15	16	17	
Emergency hospitalization	+	+	+	+	−	+	+	+	+	−	+	+	+	−	−	−	+	0.7764
History of surgery	−	+	+	+	+	−	+	+	+	+	−	+	+	−	+	+	+	0.5909
Use of inotropic agents during HD only	+	−	−	−	+	+	−	−	−	−	−	+	−	−	−	−	−	0.5721
Pre‐HD diastolic blood pressure (first session) [mmHg]	79	91	82	59	73	80	92	86	78	48	55	52	84	83	54	85	86	0.4947
BMI	26.8	21.6	29.4	20.1	18.5	14.2	21.2	24.5	20.2	19.6	25.9	15.6	25.2	18	24.8	18.6	22.7	0.4924
Lymphocyte percentage [%] at admission	12.9	12.7	0	7.6	21	25.8	1	1.4	10.2	16.2	10.2	3	2	98	26	60	4	0.4908
Past history of liver failure	−	−	−	−	−	−	−	−	−	+	+	−	−	−	+	−	−	0.4682
Pre‐HD heart rate (first session) [bpm]	85	95	85	63	88	73	99	110	78	92	101	89	102	84	73	95	67	0.4093
HFNC during HD	−	−	−	−	−	−	−	−	−	−	−	−	−	−	−	−	−	−0.0655
Presence of device: S‐ICD	−	−	−	−	−	−	−	−	−	−	−	−	−	−	−	−	−	−0.0782
History of mechanical circulatory support: others	−	−	−	−	−	−	−	−	−	−	−	−	−	−	−	−	−	−0.1276
Past history of pancreatitis	−	−	−	−	−	−	−	−	−	−	−	−	−	−	−	−	−	−0.1492
Mechanical ventilation during HD	−	−	−	−	−	−	−	−	−	−	−	−	−	−	−	−	−	−0.1717
Presence of HIV infection	−	−	−	−	−	−	−	−	−	−	−	−	−	−	−	−	−	−0.1836
Treatment setting: ECUM	−	−	−	−	−	−	−	−	−	−	−	−	−	−	−	−	−	−0.2546
Presence of device: CRT‐D	−	−	−	−	−	−	−	−	−	−	−	−	−	−	−	−	−	−0.2902
Presence of device: PM	−	−	−	−	−	−	−	−	−	−	−	−	−	−	−	−	−	−0.3162
History of mechanical circulatory support: IABP	−	−	−	−	−	−	−	−	−	−	−	−	−	−	−	−	−	−0.3321
Pre‐HD systolic blood pressure (first session) [mmHg]	124	133	140	127	159	148	153	115	132	71	109	99	145	141	104	130	139	−0.3543
History of mechanical circulatory support: ECMO	−	−	−	−	−	−	−	−	−	−	−	−	−	−	−	−	−	−0.3853
Vascular access: AVG	−	−	−	−	−	−	−	−	−	−	−	−	−	−	−	−	−	−0.477
History of maintenance HD [years]	0	0	0	13.3	2	5.3	0	0	0	1.8	0	2	0	0.3	0	1.3	0	−0.6658
Dry weight [kg]	70	52	73	56	40.6	31	46	52.9	43	56.6	62.2	37	71	41.5	54	42	56	−0.9717
*p* (ocurrence|z)	0.18	0.14	0.02	0	0.25	0.32	0.11	0.17	0.09	0.16	0.23	0.44	0.03	0.21	0.25	0.22	0.03	

Abbreviations: AUROC, area under the receiver operating characteristic curve; AVG, arteriovenous graft; BMI, body mass index; CRT‐D, cardiac resynchronization therapy with defibrillator; ECMO, extracorporeal membrane oxygenation; ECUM, extracorporeal ultrafiltration; HD, hemodialysis; HFNC, high‐flow nasal cannula; IABP, intra‐aortic balloon pump; ICD, implantable cardioverter‐defibrillator; PM, pacemaker; S‐ICD, subcutaneous ICD.

## 4 Discussion

In this study, we developed and validated a predictive model for sudden complications during HD using 23 pre‐HD covariates, achieving high predictive performance with an area under the receiver operating characteristic curve (auROC) of 0.889.

Few previous studies have investigated machine learning–based risk prediction models for clinically unstable and heterogeneous high‐risk inpatients receiving HD, such as those admitted to ICUs in acute care settings. Our model successfully identified pre‐dialysis variables predictive of adverse events in this population and may support early screening and intervention strategies.

IDH is one of the most common complications among patients undergoing maintenance HD and is associated with increased cardiovascular and all‐cause mortality [7]. Although cardiac arrest during HD is relatively rare, approximately 60% of patients who experience such events die within 48 h, and 13% of these deaths occur during the dialysis session itself [3]. In our study, two of the three patients who underwent cardiopulmonary resuscitation (CPR) died within 72 h after HD, underscoring CPR as a critical indicator of poor prognosis.

Several studies have applied machine learning techniques to predict IDH; however, most have focused on outpatient populations [9, 10, 11, 12, 13], and relatively few have addressed the prediction of other severe complications. Although existing deep learning models have demonstrated high predictive performance, they often exclude clinically complex inpatients with conditions such as sepsis, multi‐organ failure, or postoperative states. Our study addresses this gap by specifically targeting a high‐risk inpatient population marked by clinical instability and substantial heterogeneity.

Following risk factor screening using machine learning techniques, 23 covariates associated with the highest auROC were selected. In terms of clinical relevance, previous studies have identified dry weight, pre‐HD blood pressure, pre‐HD heart rate, and lymphocyte count as consistent predictors of IDH, as also reported by Hong et al. [13]. Additional covariates such as recent emergency hospitalization or surgery and shorter HD history were associated with increased risk of sudden events. Although longer HD duration has been linked to a higher overall incidence of IDH [14, 15], the cumulative risk of sudden deterioration may increase with frequent IDH episodes.

Importantly, our model targeted inpatients rather than stable outpatients. Many of the target patients had recently initiated HD or were clinically unstable due to recent surgery or emergency admission, with nearly half having a history of ICU admission. High mortality rates have been reported among patients who underwent their first hemodialysis for conditions such as AKI in the ICU [16, 17].

In this context, other covariates such as the use of inotropic agents during HD or a history of liver failure were also reasonable predictors of high risk.

The relationship between blood pressure and outcomes in patients receiving HD is known to be U‐shaped. Mortality is lowest within a systolic blood pressure (SBP) range of 140–160 mmHg and a diastolic blood pressure (DBP) range of 65–75 mmHg [18]. Hypotension is often linked to cardiac dysfunction and malnutrition; therefore, lower SBP may increase risk. Conversely, elevated DBP may indicate fluid overload, also contributing to risk [19]. Heart rate (HR) has similarly been associated with outcomes; a pre‐HD HR above 80 bpm is linked to increased mortality [20]. In terms of body mass index (BMI), higher BMI has been correlated with greater mortality risk, with a tendency toward more frequent cardiac and cerebrovascular deaths [21, 22]. An elevated HR may reflect compensatory responses to cardiac dysfunction, hypovolemia, or systemic inflammation, thereby increasing the likelihood of fatal arrhythmias and IDH.

An increased lymphocyte percentage may reflect active infection or chronic inflammation, both of which heighten the risk of sudden deterioration [23]. However, in our study, the lymphocyte percentage in the sudden event group was not significantly higher than in the non‐sudden group.

Interestingly, the use of high‐flow nasal cannula (HFNC), mechanical ventilation, or cardiac implantable electronic devices (CIEDs) during HD was associated with a decreased risk of acute events in our model. These interventions may help stabilize respiratory and cardiovascular functions, thereby lowering the likelihood of complications.

Additionally, a history of mechanical circulatory support (MCS) was also linked to reduced risk. Although the in‐hospital mortality rate for patients receiving MCS remains high (46%), survivors are typically younger, experience less severe shock, and require less invasive support [24, 25].

Although some patients develop hypotension due to increased ultrafiltration volumes, prior studies have demonstrated that early fluid management is significantly associated with reduced short‐term mortality in chronic hemodialysis patients requiring continuous renal replacement therapy in the ICU [26]. In our cohort, many patients received appropriate fluid management during the acute phase of ICU care, which may have contributed to a reduction in adverse outcomes. These observations support the clinical relevance of the covariates selected for our model and underscore the robustness of the applied feature selection process.

While few studies have focused on identifying risk factors in clinically unstable and heterogeneous high‐risk inpatient populations—such as those with a history of ICU or emergency admission—our model successfully extracted 23 preoperative variables with strong predictive performance (auROC: 0.889). Notably, several of these variables are potentially modifiable, offering opportunities for proactive screening and early intervention. Previous research has shown that outcomes following in‐hospital cardiac arrest in maintenance dialysis patients are comparable to those of nondialysis patients, indicating that CPR is not a futile intervention in this population [27]. These findings highlight the importance of enhanced monitoring and risk assessment for high‐risk hemodialysis patients to improve clinical outcomes.

Furthermore, the ability to identify high‐risk patients in advance may not only enhance patient safety by enabling timely clinical interventions but also support more efficient use of healthcare resources. This includes optimizing staffing allocation and managing clinical workloads more effectively [28].

Our model can estimate the risk of abrupt deterioration during HD using only 23 pre‐HD covariates.

## 5 Limitations

This study had several limitations. First, this was a single‐center, retrospective study. Second, the dataset primarily consisted of severely ill inpatients with prior surgical or ICU admissions, which may limit the generalizability of the findings to a broader outpatient HD population. Third, we selected logistic regression due to its interpretability and ease of implementation within clinical systems—both critical for transparent clinical decision‐making. While logistic regression served as a strong and interpretable baseline, future studies may explore the use of advanced algorithms such as XGBoost. XGBoost has demonstrated high predictive performance in various medical applications by effectively modeling nonlinear relationships and handling missing data [29]. However, its lower inherent interpretability necessitates the use of explainability tools such as SHAP to ensure clinical transparency and maintain trust in model‐driven decisions [30].

## 6 Conclusion

In the present study, we developed and validated a predictive model for sudden HD complications. The model utilized 23 covariates that could be obtained before the initiation of HD sessions, enabling the prediction of adverse events. This approach facilitates the early identification of high‐risk patients and supports the implementation of timely interventions, potentially enhancing the overall safety of HD.

## Author Contributions

Conceptualization: K.H., C.O.(Chikara Ohyama), T.G., T.O., N.K.; Data curation: T.O., N.K., C.O. (Chikako Ominato), K.N., K.K.; Formal analysis: Y.S.; Investigation: T.O., N.K., C.O. (Chikako Ominato), K.N., K.K.; Methodology: Y.S.; Project administration: K.H., C.O. (Chikara Ohyama), T.G., T.O., N.K.; Software: Y.S.; Statistical analysis: Y.S., N.K.; Supervision: K.H., C.O. (Chikara Ohyama), S.H., H.K., T.G.; Visualization: Y.S., S.H., N.K.; Writing – Original draft: N.K.; Writing – review and editing: K.H., C.O. (Chikara Ohyama), S.H., H.K., T.G. All the authors have read and agreed to the published version of the manuscript.

## Ethics Statement

This study was approved by the Ethics Committee of Hirosaki University Graduate School of Medicine (approval number: 2024–029).

## Conflicts of Interest

Kazuyoshi Hirota received a Grant‐in‐Aid for Scientific Research from the Japan Society for the Promotion of Science and speaking honoraria from Mundipharma K.K. In addition, Kazuyoshi Hirota's department received donations for research from Towada City Hospital. Shingo Hatakeyama received honoraria from Janssen Pharmaceutical K.K., Astellas Pharma Inc., AstraZeneca K.K., Ono Pharmaceutical Co. Ltd., Bayer AG, Pfizer Inc., Bristol‐Myers Squibb, Merck Biopharma Co. Ltd., Kaneka Corporation, and Nipro Corporation. The other authors declare no conflicts of interest.

## Data Availability

The data presented in this study are available upon reasonable request from the corresponding author. The data are not publicly available owing to privacy and ethical restrictions.
